# Amino Acid Substitutions of MagA in *Klebsiella pneumoniae* Affect the Biosynthesis of the Capsular Polysaccharide

**DOI:** 10.1371/journal.pone.0046783

**Published:** 2012-10-31

**Authors:** Tzu-Lung Lin, Feng-Ling Yang, An-Suei Yang, Hung-Pin Peng, Tsung-Lin Li, Ming-Daw Tsai, Shih-Hsiung Wu, Jin-Town Wang

**Affiliations:** 1 Department of Microbiology, National Taiwan University College of Medicine, Taipei, Taiwan; 2 Institute of Biological Chemistry, Academia Sinica, Taipei, Taiwan; 3 Genomics Research Center, Academia Sinica, Taipei, Taiwan; 4 Department of Internal Medicine, National Taiwan University Hospital, Taipei, Taiwan; Université d'Auvergne Clermont 1, France

## Abstract

Mucoviscosity-associated gene A (*magA*) of *Klebsiella pneumoniae* contributes to K1 capsular polysaccharide (CPS) biosynthesis. Based on sequence homology and gene alignment, the *magA* gene has been predicted to encode a Wzy-type CPS polymerase. Sequence alignment with the Wzy_C and RfaL protein families (which catalyze CPS or lipopolysaccharide (LPS) biosynthesis) and topological analysis has suggested that eight highly conserved residues, including G308, G310, G334, G337, R290, P305, H323, and N324, were located in a hypothetical loop region. Therefore, we used site-directed mutagenesis to study the role of these residues in CPS production, and to observe the consequent phenotypes such as mucoviscosity, serum and phagocytosis resistance, and virulence (as assessed in mice) in pyogenic liver abscess strain NTUH-K2044. Alanine substitutions at R290 or H323 abolished all of these properties. The G308A mutant was severely impaired for these functions. The G334A mutant remained mucoid with decreased CPS production, but its virulence was significantly reduced *in vivo*. No phenotypic change was observed for strains harboring *magA* G310A, G337A, P305A, or N324A mutations. Therefore, R290, G308, H323, and G334 are functionally important residues of the MagA (Wzy) protein of *K. pneumoniae* NTUH-K2044, capsular type K1. These amino acids are also likely to be important for the function of Wzy in other capsular types in *K. pneumoniae* and other species bearing Wzy_C family proteins.

## Introduction

*Klebsiella pneumoniae* is an opportunistic Gram-negative bacterium that causes urinary tract infections, nosocomial pneumonia, and intra-abdominal infections [1]. A new type of invasive *K. pneumoniae* disease has emerged worldwide as the source of community-acquired pyogenic liver abscess (PLA), especially in Asia [2], [3], [4], [5]. This disease often is complicated by metastatic infections such as meningitis and endophthalmitis.

In our previous study, we had screened for mucoviscosity-associated genes by using a transposon mutant library of a *K. pneumoniae* PLA strain, NTUH-K2044. The *magA* (mucoviscosity-associated gene A) was identified based on its role in mucoviscosity, resistance to serum killing and phagocytosis, and virulence in mice [6]. Based on limited sequences similarities, MagA was proposed to be an O-antigen ligase [6]. In our subsequent work, we found that *magA*-adjacent sequences encoded proteins homologous to capsular polysaccharide (CPS) biosynthetic machinery; deletion/complementation experiments proved that *magA* was an essential gene for K1 CPS biosynthesis [7]. Therefore, the mucoviscosity is indirectly related to *magA* because of its essential role in capsule production, whereas the mucoviscosity might be mediated by capsule expression promoting regulators such as *rmpA* [8], [9].

However, the actual function of MagA in the biosynthesis of the CPS in *K. pneumoniae* remains undefined. Genetic alignment (synteny) of *cps* regions revealed that the *K. pneumoniae* loci could be classified as group 1. Characterization of capsular biosynthesis in *Escherichia coli* demonstrated that the export and polymerization of group-1 CPS is controlled by a Wzy (polymerase)-dependent system [10], [11]. Specifically, undecaprenolpyrophosphoryl-linked repeat units of CPS are assembled in the cytoplasm, transferred across the plasma membrane by a flippase Wzx [12], and polymerized in the periplasmic space by a Wzy polymerase [13]. The mature CPS then is translocated and exported to the bacterial surface through the combined action of an inner membrane tyrosine autokinase (Wzc), a low-molecular-weight protein-tyrosine phosphatase (Wzb), and an integral outer membrane lipoprotein (Wza) [10], [14]. Recently, amino-acid sequence comparison and domain conservation suggested that MagA is a Wzy polymerase [15], [16]. The role of *magA* in O-antigen biosynthesis has been excluded and the involvement of *magA* in K1 CPS biosynthesis has been confirmed again [16]. In this study, we identified the conserved amino acids of MagA and used Ala substitutions to analyze the role of these residues in CPS biosynthesis in the PLA strain NTUH-K2044.

## Materials and Methods

### Bacterial strains and culture conditions

*K. pneumoniae* mutants were constructed in the NTUH-K2044 strain background. *E. coli* DH10B was used for standard cloning and plasmid construction. Both *K. pneumoniae* and *E. coli* were grown in Luria-Bertani (LB) broth or agar at 37°C, except as noted below. Where appropriate, medium was supplemented with kanamycin (50 µg/mL) or sucrose (5%).

### Site-directed mutagenesis of *magA*

Plasmid pCRII-TOPO-*magA*-CAT [6] was used as a template. The indicated (underlined) codon was mutated to encode Ala by using one of the following primers and high-fidelity Pfu DNA polymerase (Stratagene, La Jolla, CA) [17]: G308A (5′-GAGCATCCAATACTT**GCA**GAGGGAGTGGGA-3′), G310A (5′-GCATCCAATACTTGGAGAG**GCA**GTGGGAATATTC-3′), G334A (5′-CTGCAGCAGAAACG**GCA**ATTGTTGGCATATTATTAACC-3′), G337A (5′-GCAGCAGAAACGGGAATTGTT**GCC**ATATTATTAACCGT-3′), R290A (5′-CAGTCCGAAAGTGAA**GCA**ATTGATGCTTGGCATCATGC-3′), P305A (5′-CCACGTTTTATGAGCAT**GCA**ATACTTGGAGAGGG-3′), H323A (5′-GATTCAACATGTACCCT**GCT**AATATATTCTTCGAATCTGC-3′), and N324A (5′-CAACATGTACCCTCAT**GCT**ATATTCTTCGAATCTGCAGC-3′). Following temperature cycling the product was treated by the restriction enzyme DpnI, and then transformed into *E. coli* DH10B competent cells. The clones harboring desired mutation were screened by sequencing using appropriate primers.

### Site-directed mutagenesis of chromosomal *magA* and complementation

*magA* site-directed mutants were generated using a pKO3-km vector that contains a temperature-sensitive origin of replication and markers for positive and negative selection for chromosomal integration and excision [18], [19], [20]. Fragments containing mutagenized *magA* genes (generated by PCR, as above) were cloned individually into the NotI site of a pKO3-Km plasmid. The resulting constructs were then electroporated into wild type strain. The transformants were cultured at 43°C. Five colonies were picked in 1 ml LB broth followed by serial dilution and plating onto LB plates containing 5% sucrose and cultured at 30°C. Colonies were screened for kanamycin susceptibility, and chromosomal gene replacement was confirmed by PCR and sequencing using appropriate primers. These *magA* point mutants were also complemented by the pCRII-TOPO-*magA*-CAT plasmid described previously [6]. The complementation strains were selected by LB agar plates supplemented with chloramphenicol (100 µg/mL).

### Mucoviscosity

The mucoviscosity of *K. pneumoniae* was determined by a string test and measured by centrifugation as described previously [6], [21], [22]. The string test was performed by stretching a colony which was grown overnight on blood agar plate using a loop. The observation of a >5 mm string was considered as the string-test positive. To further measure the levels of mucoviscosity, a low-speed centrifugation was performed. Briefly, equal numbers of overnight-cultured bacteria were centrifuged at 1000 g for 5 min. Then, the supernatant was subjected to measurement of the absorbance at 600 nm.

### Quantitative measurement of bacterial capsular polysaccharide

The bacterial extracellular polysaccharide was extracted and the uronic acid, a main component of *K. pneumoniae* K1 capsule, was quantified as previous described [23], [24]. Briefly, 500 µl of overnight broth-cultured bacteria was mixed with 100 µl of 1% Zwittergent 3–14 (Sigma-Aldrich, Milwaukee, WI) in 100 mM citric acid (pH 2.0) and then incubated at 50°C for 20 min. After centrifugation, 250 µl of the supernatant was transferred and added with 1 ml of cold ethanol. The mixture was incubated at 4°C for 20 min for precipitation. After centrifugation, the pellet was dried and dissolved in 200 µl of distilled water, and then 1200 µl of 12.5 mM tetraborate in concentrated H_2_SO_4_ was added. After vigorous vortex, the mixture was boiled for 5 min. After cooling, 20 µl of 0.15% 3-hydroxydiphenol (Sigma-Aldrich) was added. Then, the absorbance at 520 nm was measured.

### K1 serotyping

The K1 serotyping was performed by the double immunodiffusion assay, as described previously [7]. In brief, 1 ml of overnight broth-cultured bacteria were harvested and resuspended in 150 µl water. Equal volume of hot phenol (pH6.6) was added. After vigorous vortex, the mixture was then incubated in 65°C for 20 min followed by chloroform extraction and centrifugation. Extracted polysaccharides were reacted with the K1 serotype-specific antiserum (Statens Serum Institute, Copenhagen, Denmark) using a double immunodiffusion assay. Each assay was performed with a 1.5% Nobel agar in normal saline (0.9% sodium chloride). Ten µl serum (75% dilution) was loaded into the central well and 20 µl of the polysaccharide extracts each was loaded into peripheral wells. After an overnight incubation at 37°C, the precipitation lines were read and stained with 1% Azocarmine (Chroma) dissolved in 2% glacial acid.

### Serum and phagocytosis resistance assay and animal inoculation

The serum and phagocytosis resistances of *K. pneumoniae* strains were determined as described previously [6], [21]. A total of 2.5×10^4^ CFU bacteria in 25 µl were mixed with 75 µl serum from healthy human volunteers. The mixture was incubated at 37°C for 3 hours. Then, bacterial numbers were determined and survival ratio was calculated. A survival ratio≥1 corresponds to serum resistance. *Dictyostelium discoideum* AX-2 cells were grown at 23°C in a HL5 medium. *K. pneumoniae* strains were plated on an SM agar plate, and 5,000 *Dictyostelium* cells in 2 µl of HL5 media were added on the plate center for four-day incubation at 22°C.

All animal experiments were followed the guidelines in the Handbook of Laboratory Animal Care of the National Laboratory Animal Breeding and Research Center, National Science Council of Taiwan, and were approved by the Animal Committee of the National Taiwan University College of Medicine. Animal experiments were performed as described previously [6]. Female BALB/cByl 5-week-old mice were used for inoculation of *K. pneumoniae* intraperitoneally. Four mice were used to test the effect of each inoculum. After inoculation, the mice were observed for 30 days. The LD_50_ was calculated using the method established by Reed and Muench [25].

### Statistical analysis

Statistical significance of comparisons of mean values was assessed by a two-tailed Student's *t* test using a Prism 5 (Graphpad) software. *P* values of <0.05 were considered significant.

## Results

### Identification of conserved residues of MagA

**Figure 1:**
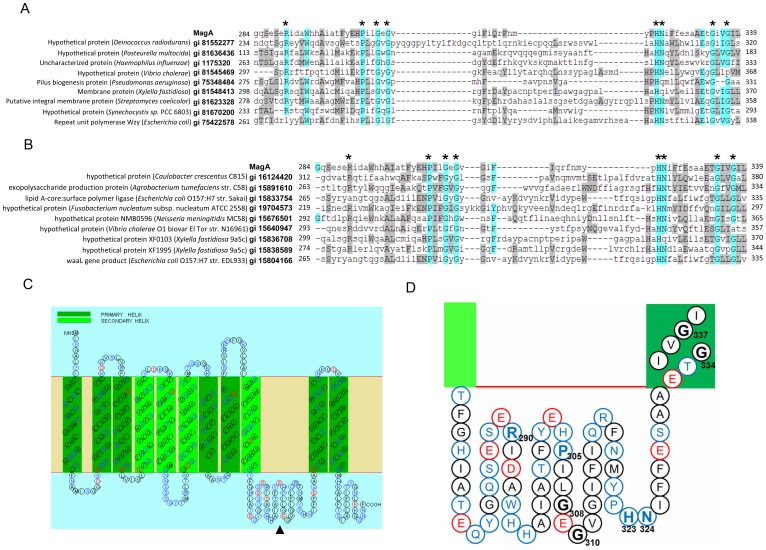
Conserved amino acids and the predicted transmembrane topology of MagA. Use MagA sequence to query NCBI Conserved Domain Database found two types of protein domains sharing the similarities with the amino-acid residues 284–339 of MagA. Eight conserved amino acids are found in both protein domain alignments. Topology prediction shows that most conserved amino acids are located in an inter-transmembrane region. **A**., **B.** The sequence alignments of MagA and nine sequences with Wzy_C domain, and nine sequences with RfaL, respectively. The sequences were aligned by the MUSCLE web tool (http://www.bioinformatics.nl/tools/muscle.html) using default parameters. Amino acids are colored in Belvu program by average similarity according to BLOSUM62 substitution matrix score from most similar (light blue) to less similar (gray). The residues showing high conservation in both profiles are marked with stars. **C.** The predicted topology of MagA made by SOSUI. The hypothetical loop (residue number 274 to 331) is indicated by the arrow head. **D.** The locations of eight conserved amino acids (a zoom of Figure 1C). Conserved amino acids are labeled by bold residue numbers. Six of eight conserved amino acids are on the loop. The other two are in the adjacent transmembrane area.

The Basic Local Alignment Search Tool (BLAST) was used to query the GenBank sequence database for proteins with similarity to MagA (accession number AB198423). The search results for conserved domains indicated that two families of protein sequences could be partially aligned with MagA. However, the alignment was limited to amino acid residues 284–339 of the MagA protein, and the sequence similarity was marginal at best. These two families are the C domain of the Wzy polymerase (Wzy_C; E-value = 1.11e-07) (Fig. 1A) and, to a lesser extent, RfaL (O-antigen ligase) (E-value = 1.37e-03) (Fig. 1B). Both protein families are related to polysaccharide (CPS or LPS) synthesis, but the synthetic mechanism of these two protein families still has not been clearly elucidated. BLAST conserved-domain alignments with MagA additionally recovered another nine sequences to Wzy_C and RfaL with high similarity, respectively (Figures 1A and 1B). Among MagA and these 18 aligned sequences, amino acid identity was observed at residues G308, G310, G334, P305, and H323. Strongly conserved residues included G337 (shared in 16 of 19 sequences), R290 (15 of 19), and N324 (15 of 19).

Three web services of predicting membrane topology, SOSUI [26], TMHMM [27] and TopPred2 [28], were used to predict the topology of MagA. The predicted topologies were shown in the Figure 1C (SOSUI) and Figure S1 (TMHMM). MagA was predicted to have 10∼11 transmembrane helices based on three predictions. The predicted transmembrane topology for MagA was similar to those of Wzy_C and RfaL [29], indicating that the sequence relationship among these proteins could extend to the level of structural similarity. The segment which was identified to share similarities with Wzy_C and RfaL domains was predicted as a consecutively inter-transmembrane sequence. Figure 1D showed the locations of conserved amino acids with the topology predicted by SOSUI. Six of eight amino acids (R290, P305, G308, G310, H323, and N324) were located on this hypothetical loop while the other two amino acids (G334 and G337) were in the N-terminal of transmembrane region which was adjacent to the hypothetical loop. Therefore, our alignments point to a total of 8 amino acids residues that were structurally shared and presumed functionally important.

### Conserved amino acid analysis in Wzy homologs of other capsular types

*cps* sequences for *K. pneumoniae* of thirteen capsular types were available in Genbank, including serotypes K1, K2, K5, K9, K14, K20, K52, K54, K57, K62, and KN1, as well as two unknown types (KPB and KPC) [6], [7], [15], [30], [31]. The putative *wzy* locus in each *cps* region was identified by BLAST-P (Table 1). Among these thirteen capsular types, a Wzy_C domain-containing Wzy homolog was identified in the *cps* of five capsular types (K1, K20, K57, K62 and KPC). The highly conserved amino acid residues identified in MagA (Wzy) of K1 also were present in those of other four capsular types (K20, K57, K62, and KPC), but in capsular types K57 and K62, the residue corresponding to H323 was aspartic acid. Additionally, the glycine corresponding to G337 appeared to be absent from the Wzy homolog of capsular type KPC. The sequences that aligned to Wzy_C domain of five capsular types each were located in a hypothetical loop region. Therefore, these conserved amino acids were expected to be important for the function of Wzy in other capsular types in *K. pneumoniae*.

**Table 1:** Analysis of putative Wzy in published *cps* sequences of *K. pneumoniae*.

capsular type	strains	putative *wzy*	nucleotide location	accession number	Wzy-C domain (a.a. location)	conservative amino-acid residues
K1	NTUH-K2044	*magA*	17813–19039	AB198423	+(204–340)	R290, P305, G308, G310, H323, N324, G334, G337
	DTS	*magA*	12908–14134	AY762939	+(204–340)	R290, P305, G308, G310, H323, N324, G334, G337
K2	Chedid	*orf10*	13905–15194	D21242	−	
	VGH525	*orf10*	11633–12922	AB371296	−	
K5	NTUH-K9534	*wzy_K5*	8516–9727	AB289646	−	
	Kauffmann E5051	*wzy*	8500–9711	AB289645	−	
	VGH404	*kp5A*	8365–9576	AB371292	−	
K9	VGH484	*kp9D*	11839–13011	AB371293	−	
K14	VGH916	*kp14E*	12819–14027	AB371294	−	
K20	NTUH-KP13	*wzy_K20*	10309–11160	AB289648	+(51–214)	R139, P155, G158, G160, H195, N196, G206, G209
	889/50	*wzy*	12441–13292	AB289647	+(51–214)	R139, P155, G158, G160, H195, N196, G206, G209
	NK8 (KPA)	*wzy*	9777–11009	AB371289	+(178–341)	R266, P282, G285, G287, H322, N323, G333, G336
K52	MGH78578	KPN_02503	2732856–2733980	NC_009648	−	
K54	NTUH-KP35	*wzy_K54*	10918–12000	AB289650	−	
K57	A1142	*wzy*	13440–14618	AB334776	+(208–337)	R285, P300, G303, G305, D318, N319, G329, G332
K62	VGH698	*kp62E*	13043–14209	AB371295	+(201–328)	R281, P290, G293, G295, D306, N307, G320, G323
KN1	A1517	*orf12*	14499–15671	AB334777	−	
KPC	NK245	*kpc8*	16994–18271	AB371291	+(213–351)	R294, P309, G312, G314, H338, N339, G349
KPB	NK29	*kpb2*	9742–11040	AB371290	−	

### Mucoviscosity of site-directed *magA* point mutants

**Figure 2:**
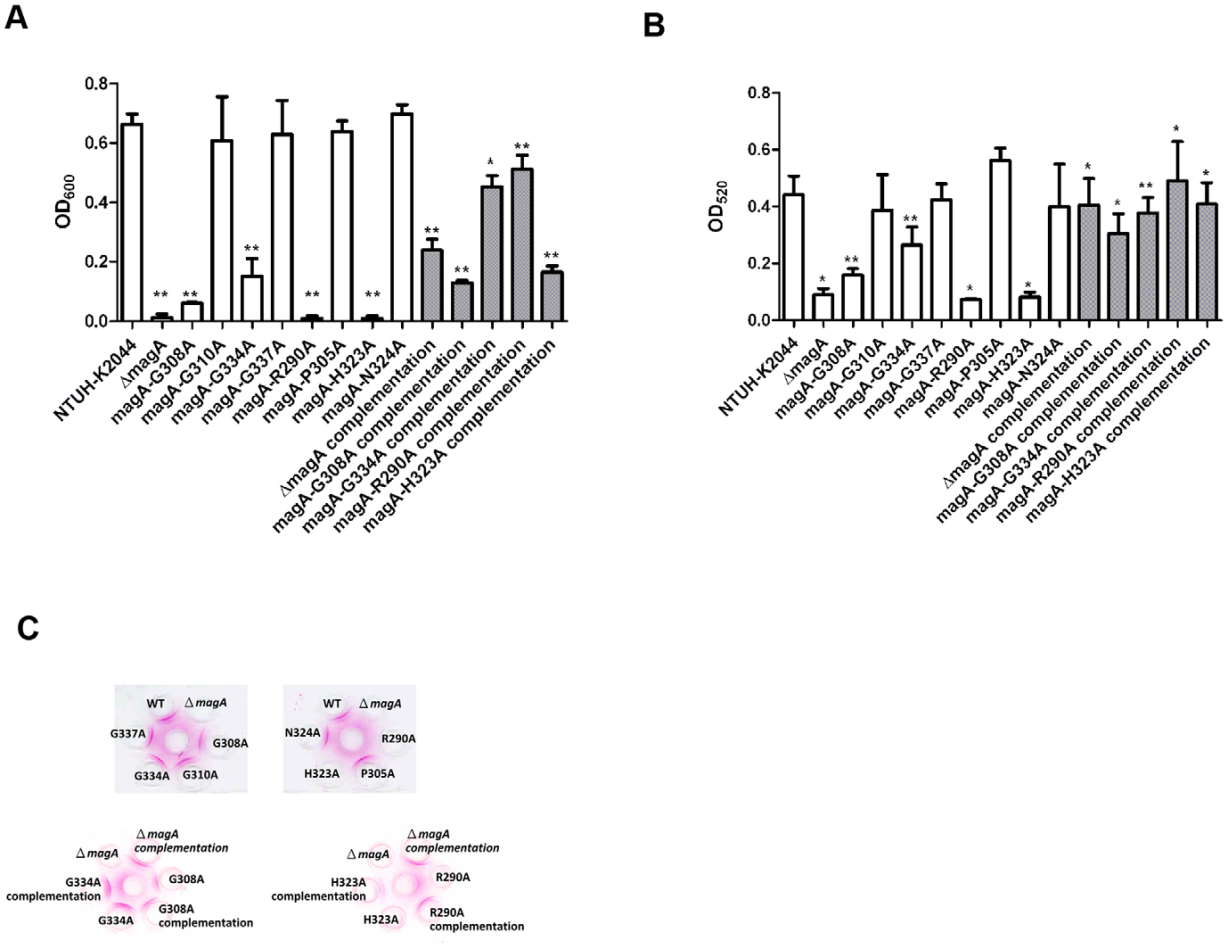
Mucoviscosity and CPS of *magA* point mutants. **A.** Mucoviscosity of *K. pneumoniae* NTUH-K2044 wild type, Δ*mag*A mutant, *mag*A (G308A, G310A, G334A, G337A, R290A, P305A, H323A and N324A) site-directed mutant strains and complementation strains. The mucoviscosity determined by centrifugation was represented by OD_600_ of three independent experiments (mean±SD). (wild type vs. Δ*mag*A, *P*<0.0001; wild type vs. *mag*A-G308A, *P* = 0.0012; wild type vs. *mag*A-G334A, *P* = 0.0097; wild type vs. *mag*A-R290A, *P* = 0.0012; wild type vs. *mag*A-H323A, *P* = 0.0012; Δ*mag*A vs. Δ*mag*A complementation, *P* = 0.0070; *mag*A-G308A vs. *mag*A-G308A complementation, *P* = 0.0034; *mag*A-G334A vs. *mag*A-G334A complementation, *P* = 0.0181; *mag*A-R290A vs. *mag*A-R290A complementation, *P* = 0.0029; *mag*A-H323A vs. *mag*A-H323A complementation, *P* = 0.0031; Student's *t* test) **P<0.01 *P<0.05. **B**. CPS production of *K. pneumoniae* NTUH-K2044 wild type, Δ*mag*A mutant, *mag*A (G308A, G310A, G334A, G337A, R290A, P305A, H323A and N324A) site-directed mutant strains and complementation strains. The amount of CPS was represented by OD_520_ of three independent experiments (mean±SD). (wild type vs. Δ*mag*A, *P* = 0.02; wild type vs. *mag*A-G308A, *P* = 0.0098; wild type vs. *mag*A-G334A, *P* = 0.0048; wild type vs. *mag*A-R290A, *P* = 0.0109; wild type vs. *mag*A-H323A, *P* = 0.0173; Δ*mag*A vs. Δ*mag*A complementation, *P* = 0.0392; *mag*A-G308A vs. *mag*A-G308A complementation, *P* = 0.0332; *mag*A-G334A vs. *mag*A-G334A complementation, *P* = 0.0040; *mag*A-R290A vs. *mag*A-R290A complementation, *P* = 0.0349; *mag*A-H323A vs. *mag*A-H323A complementation, *P* = 0.0239; Student's *t* test) **P<0.01 *P<0.05. **C.** Double immunodiffusion assays of rabbit anti-*K. pneumoniae* K1 serum with capsular extracts of different *K. pneumoniae* strains. The rabbit anti-K1 serum from Statens Serum Institute is in the center well and capsular extracts of overnight-cultured *K. pneumoniae* strains are in peripheral wells. The names of the strains are labeled in the peripheral wells. A red precipitation line near the peripheral well is considered as a positive reaction.

In order to clarify the function of these conservative amino acids, we generated *magA* point mutants in which each of these eight conserved residues was separately substituted with alanine. Alanine substitution is a rapid way to determine the contribution of a specific amino acid residue to the function of the protein and does not change the main-chain conformation or other steric effects [32]. Strains harboring the G308A, R290A or H323A mutations became negative for the string test (an assay of mucoviscosity); strains harboring G310A, G334A, G337A, P305A, or N324A remained string test-positive, like the parent (data not shown). To further measure the levels of mucoviscosity, a low-speed centrifugation was performed. As shown in Figure 2A, wild-type NTUH-K2044 was highly mucoid, whereas *magA* deletion mutant became non-mucoid. Consistent with the results of the string test, the R290A and H323A mutants were non-mucoid, with a phenotype similar to that of a *magA* null (deletion) mutant; the G310A, G337A, P305A, and N324A mutants were mucoid, consistent with the appearance of the wild-type parent. Notably, although G334A was string test-positive, the mucoviscosity of this mutant was significantly reduced by the centrifugation assay. In contrast, the G308A mutation rendered the strain string test-negative but the mucoviscosity (by centrifugation) was not reduced to the level seen in the *magA* deletion mutant. Therefore, the subtle difference of mucoviscosity could be detected by low-speed centrifugation. These *magA* point mutants were complemented by the pCRII-TOPO-*magA*-CAT plasmid described previously [6]. The mucoviscosities of G308A, G334A, R290A and H323A complementation strains were significantly restored.

### Characterization of CPS in *magA* point mutants

The CPS of *K. pneumoniae* NTUH-K2044 wild type and its *magA* point mutants were quantified (Figure 2B). The strains harboring the G310A, G337A, P305A and N324A mutations (which exhibited similar mucoviscosity to the wild type strain) showed no significant difference in the amount of CPS compared with the wild type strain. The CPS productions of R290A and H323A mutants which showed non-mucoid phenotype similar to the *magA* deletion mutant were also reduced to the level seen in the *magA* deletion mutant. The G308A and G334A mutants with partial mucoviscosity showed partial decreases in the CPS production correspondingly. Overall the levels of CPS production (Figure 2B) correlate semi-quantitativley with the mucoviscosity (Figure 2A) in these *magA* point mutants. These results suggested that low-speed centrifugation is a better method than string test for detecting the bacterial mucoviscosity and CPS production. The extracelluar polysaccharides of these mutants were probed with anti-K1 antisera using double immuno-diffusion. As shown in Figure 2C, the R290A and H323A mutants which lost the CPS production also lost K1 antigenicity. For both mutants, complementation with episomal *magA* significantly restored the K1 CPS. Although the G308A and G334A mutants produced reduced amounts of CPS, the remaining CPS could still react to anti-K1 antisera.

### Serum and phagocytosis resistance and animal infection

**Figure 3:**
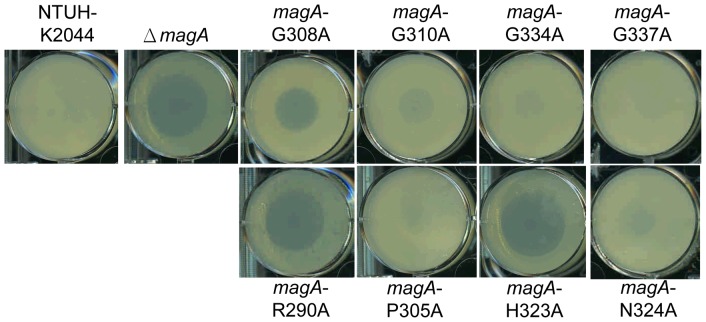
Phagocytosis resistance of *magA* point mutants. Phagocytosis resistance of *K. pneumoniae* NTUH-K2044 wild type, Δ*mag*A mutant, *mag*A (G308A, G310A, G334A, G337A, R290A, P305A, H323A and N324A) site-directed mutant strains. *K. pneumoniae* was plated on an SM agar plate, and 5,000 *Dictyostelium* cells in 2 µl of HL5 media were added on the plate center for four-day incubation at 22°C. The formation of phagocytic plaques are observed in Δ*mag*A, G308A, R290A and H323A mutants.

To further analyze the biological effects of these point mutations, *in vitro* serum and phagocytosis resistance and *in vivo* virulence in mice were determined. In contrast to the parent strain, the G308A, G334A, R290A, and H323A mutants were serum sensitive (Fig. S2). Complementation with *magA* significantly restored the serum resistance of these four point mutants. Although the survival ratios of the other four point mutants were significantly reduced, they still remained serum resistant. A recent study reported the use of phagocytosis by *Dictyostelium discoideum* as a surrogate assay to identify the phagocytosis-sensitive mutants of *K. pneumoniae in vitro* [21]. The susceptibility to *Dictyostelium* was consistent with that to human neutrophils. As shown in Figure 3, the R290A and H323A mutations rendered the bacteria as phagocytosis-sensitive as deletion of *magA*. The G308A mutant also displayed increased sensitivity to *Dictyostelium*, but not to levels seen in the *magA* deletion mutant.

We further assessed the virulence in mice of the G308A, G334A, R290A and H323A mutants. The LD_50_ of NTUH-K2044 in BALB/cByl mice was <10^2^ colony-forming units (CFU), consistent with the results of our previous study [6]. The LD_50_ values of the R290A and H323A mutants increased significantly to >10^7^ CFU, which is the same as the value derived for the *magA* transposon knockout mutant [6]. The LD_50_ values of the G308A and G334A mutants also were increased, to 3×10^6^ CFU and 2×10^6^ CFU, respectively.

Taken the above results together (Table 2); alanine substitutions at R290 or H323 abolished all of these properties including mucoviscosity, CPS production, serum and phagocytosis resistance, and virulence in mice. The G308A mutant was severely impaired for these functions. The G334A mutant remained mucoid with decreased CPS production. However, the virulence of the G334A mutant was significantly reduced *in vivo*. No phenotypic change was observed for strains harboring *magA* G310A, G337A, P305A, or N324A mutations. Overall the levels of CPS production correlate with the observed phenotypes including mucoviscosity, serum and phagocytosis resistance, and virulence in mice in these *magA* point mutants. Therefore, R290, G308, H323, and G334 are functionally important residues of the MagA protein of *K. pneumoniae* NTUH-K2044, capsular type K1.

**Table 2:** The phenotypic characterizations of *magA* point mutants.

	mucoviscosity		CPS production (ratio)*	K1	serum resistance (resistant or sensitive to 75% serum)	phagocytosis resistance (resistant or sensitive to 5000 *Dictyostelium* cells)	virulence in mice (LD_50_, cfu)
	by string test	by centrifugation (ratio)*					
NTUH-K2044 wild-type	+	1	1	+	resistant	resistant	<10^2^
Δ*magA*	−	0.018#	0.204#	−	sensitive	sensitive	>10^7^
G308A	−	0.092#	0.359#	+	sensitive	sensitive	3×10^6^
G310A	+	0.916	0.876	+	resistant	resistant	ND
G334A	+	0.227#	0.601#	+	sensitive	resistant	2×10^6^
G337A	+	0.948	0.959	+	resistant	resistant	ND
R290A	−	0.015#	0.166#	−	sensitive	sensitive	>10^7^
P305A	+	0.962	1.273	+	resistant	resistant	ND
H323A	−	0.015#	0.185#	−	sensitive	sensitive	>10^7^
N324A	+	1.052	0.906	+	resistant	resistant	ND

* comparison based on the wild type strain.

# with significant difference.

ND, not determined.

## Discussion

K1 CPS is considered as one of the essential virulent determinants for the development of invasive liver abscess [7], [33]. The *magA* locus has been shown to be involved in the CPS biosynthesis of *K. pneumoniae* serotype K1 [6], [7]. Because we were unable to produce adequate amounts of recombinant MagA protein (data not shown) to perform *in vitro* enzyme assays, the function of MagA in CPS biosynthesis could not be demonstrated directly. Recently, MagA was predicted to be a Wzy-like polymerase based on sequence homology, conserved domain alignment, and an O-antigen synthesis assay [15], [16]. Based on syntenic alignments of *cps* regions, the CPS biosynthetic machinery in *K. pneumoniae* was classified as a group 1 complex. The well-established model for understanding of group 1 bacterial Wzy-dependent CPS biosynthesis is the *E. coli* system. Specifically, in the absence of *wzy*, the high-molecular-weight capsule of *E. coli* (O9a:K30) is eliminated [13]. These results are in agreement with the phenotype of the *K. pneumoniae magA* knockout. In this study, based on predicted folding topology and sequence homology of MagA, we also judged that MagA function could be similar to that of Wzy. In addition, the sequence alignments revealed that similarity is particularly significant in the hypothetical loop region. We propose that the highly conserved amino acid residues in this loop are important for the protein's biochemical function.

Alanine substitution is a rapid way to determine the contribution of a specific amino acid residue to the function of the protein and does not change the main-chain conformation or other steric effects. Therefore, we choose this method to identify important amino acids rapidly. Targeted single Ala substitutions revealed that point mutations of G308A, G334A, R290A, or H323A was sufficient to disrupt MagA function, and the involvements of G310, G337, P305 and N324 in MagA function were excluded. The substitutions of these amino acids might change the predicted loop structure in MagA. These identified amino acids then could be further studied by less critical substitutions. The HMM logos analysis of Wzy_C domain provided in the Pfam website (http://pfam.sanger.ac.uk/family/PF04932#tabview=tab3) revealed that these four important residues of MagA (R290, G308, H323 and G334) also were the best conserved residues of the Wzy_C family. Therefore, the results in this study are expected to apply to Wzy homologs of other *K. pneumoniae* capsular types and to other Wzy_C family proteins.

*K. penumoniae* with R290A or H323A mutant of MagA lose the function for CPS biosynthesis while G308A and G334A mutants reduce the activity of MagA. Based on topological analysis of MagA, R290, G308 and H323 are located on the loop region whereas G334 is located on the transmenbrane region adjacent to the loop. Therefore, this hypothetical loop and these important amino acid residues might participate in the interaction with carbohydrate which is the main function of Wzy. In previous study of analyzing protein-carbohydrate binding sites properties [34], propensities of Arg and His in the carbohydrate binding sites are 0.5 and 1.4, respectively. The positive propensities indicate that Arg and His are preferred in the binding sites. When they are mutated to Ala, the propensity reduces to −0.48 which is not preferred in carbohydrate binding site and therefore may terminate the protein function. This result was coherent to the observations of R290A and H323A mutants. However, two wild type sequences (identical sequences from two *E. coli* O157:H7 strains) with RfaL conserved domain (Figure 1B) showed the R290A amino acid substitution. There might be different mechanisms for carbohydrate binding in this protein of these two bacteria. In the G308A and G334A mutants, since the preference of Gly in carbohydrate binding sites is less than 0.02, these two mutations are less likely participating in the carbohydrate interactions. The phenomenon shows that the CPS productions in these mutants are reduced but not destroyed supports the hypothesis described above. Therefore, mutating of these two Gly may produce structure defects and reducing the efficiency of MagA.

It has been known that CPS is important for virulence. The results in this study also revealed that the levels of CPS production correlate with the observed consequent phenotypes including mucoviscosity, serum and phagocytosis resistance, and virulence in mice. Our results of these varied assays in *magA* point mutants were consistent in revealing that R290A and H323A mutations abolished MagA function, while G308A mutation reduced but did not eliminate function. In G334A mutant, the reduced CPS production resulted in deficiency of serum resistance and decreased virulence in mice. But the phagocytic plaque was not observed in G334A mutant by adding 5000 *Dictyostelium* cells. The reduction of CPS production in G334A mutant is less than that of G308A mutant, therefore, the formation of phagocytic plaque might be observed by adding more *Dictyostelium* cells. These results not only identified important conserved residues of MagA (Wzy), but also confirmed the role of MagA in CPS biosynthesis.

In conclusion, based on topology analysis and sequence alignment, eight highly conserved amino acid residues of MagA (including R290, P305, G308, G310, H323, N324, G334, and G337) have been identified in a shared hypothetical loop region of the protein. Among these eight candidates, R290, G308, G334, and H323 are determined to be functionally important for CPS synthesis and for *in vitro* and *in vivo* virulence of the PLA strain.

## Supporting Information

Figure S1 The membrane topology prediction made by TMHMM. The arrow head indicated a consecutive region (residue number 269–333) which is corresponding to the aligned segment in Figure 1A and 1B.
(TIF)

Figure S2 Serum resistance of *magA* point mutants. Serum resistance of *K. pneumoniae* NTUH-K2044 wild type, Δ*mag*A mutant, *mag*A (G308A, G310A, G334A, G337A, R290A, P305A, H323A and N324A) site-directed mutant strains and complementation strains. The serum resistance was represented by survival ratio of three independent experiments (mean±SD). The average survival ratio≥1 corresponds to serum resistance. (comparing mutants vs. wild type strains or the complementation strains vs. mutants; Student's *t* test) **P<0.01 *P<0.05.
(TIF)
