# Supplementary figures and images for: Amino Acid Substitutions of MagA in Klebsiella pneumoniae Affect the Biosynthesis of the Capsular Polysaccharide
Source: PLoS One. 2012 Oct 31;7(10):e46783. doi: 10.1371/journal.pone.0046783 (PMC3485256; doi:10.1371/journal.pone.0046783)

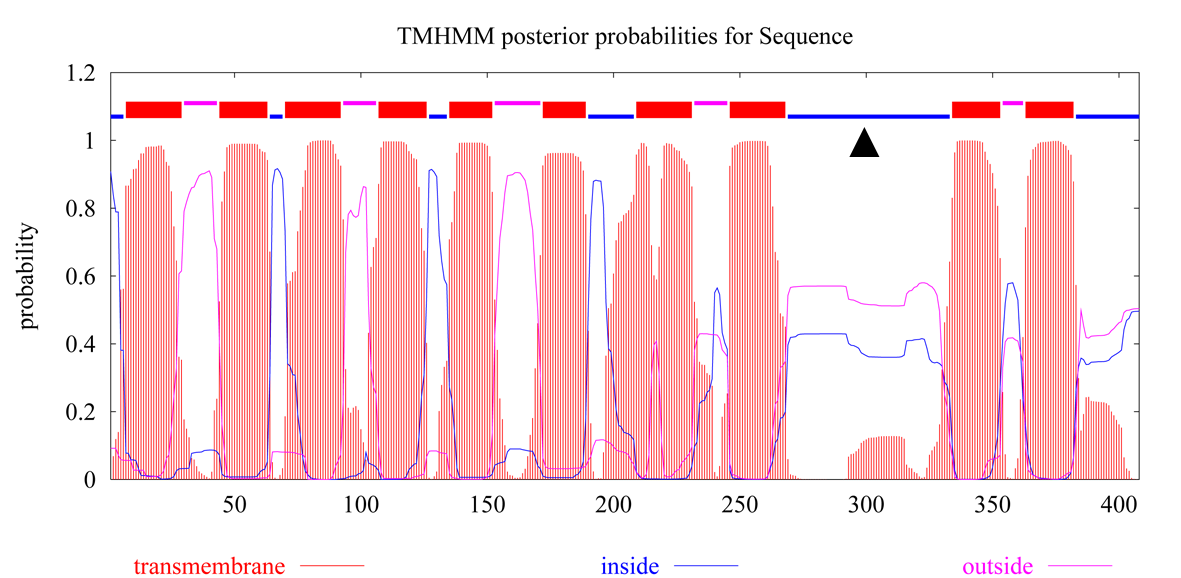

Supplement: Figure S1 — The membrane topology prediction made by TMHMM. The arrow head indicated a consecutive region (residue number 269–333) which is corresponding to the aligned segment in Figure 1A and 1B. (TIF) [file pone.0046783.s001.tif]

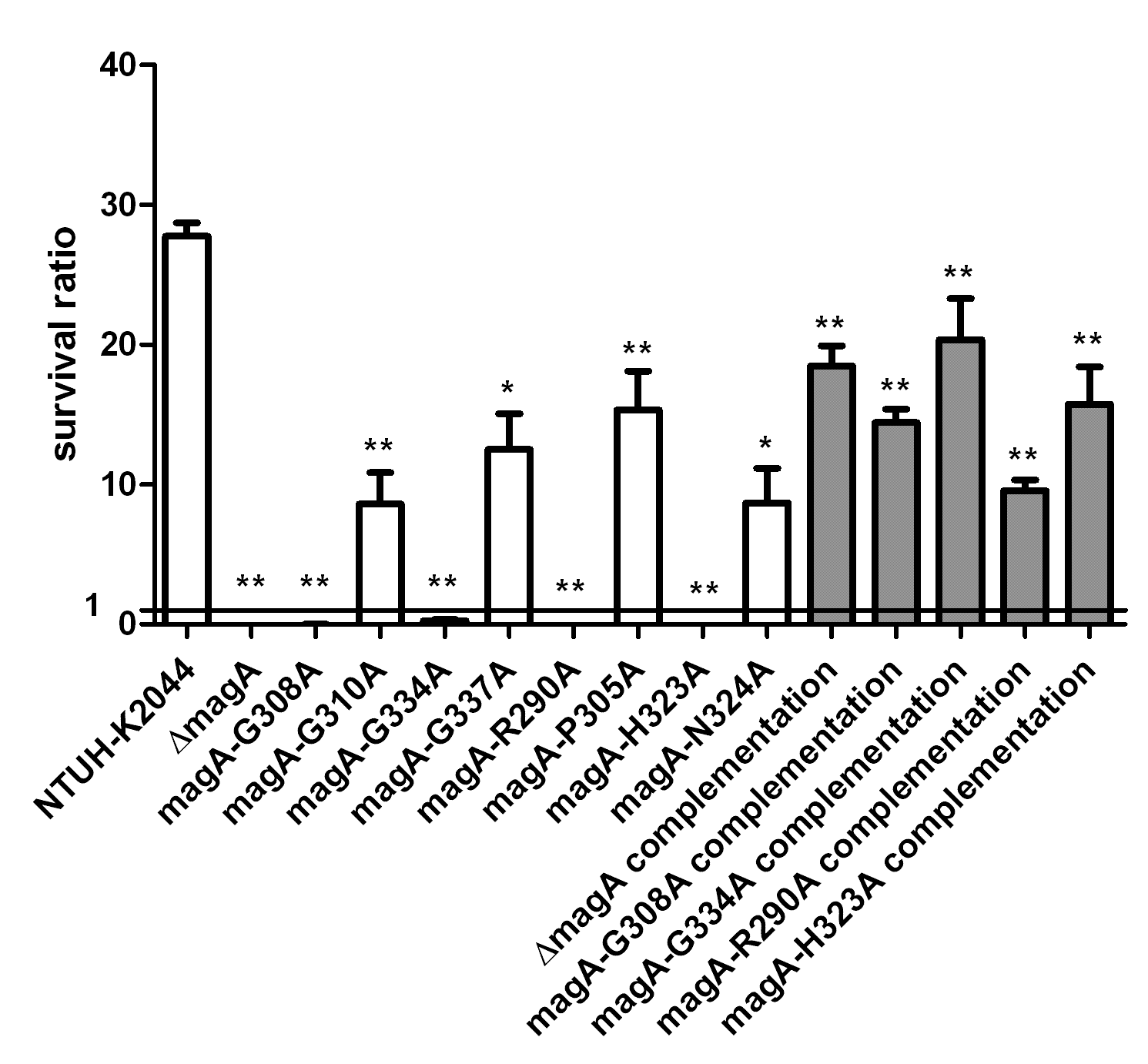

Supplement: Figure S2 — Serum resistance of magA point mutants. Serum resistance of K. pneumoniae NTUH-K2044 wild type, ΔmagA mutant, magA (G308A, G310A, G334A, G337A, R290A, P305A, H323A and N324A) site-directed mutant strains and complementation strains. The serum resistance was represented by survival ratio of three independent experiments (mean±SD). The average survival ratio≥1 corresponds to serum resistance. (comparing mutants vs. wild type strains or the complementation strains vs. mutants; Student's t test) **P<0.01 *P<0.05. (TIF) [file pone.0046783.s002.tif]
